# Effect of resveratrol supplementation on lipid metabolism in healthy and obese cats

**DOI:** 10.3389/fvets.2025.1565367

**Published:** 2025-04-16

**Authors:** Jung Eun Yun, Seung Rok Kang, Jae Young Kim, Hyun Joong Kim, Motoo Kobayashi, Toshiro Arai

**Affiliations:** ^1^Momo Group Inc, Seongnam-si, Republic of Korea; ^2^CORbio, Seoul, Republic of Korea; ^3^Seijo Kobayashi Veterinary Clinic, Setagaya, Tokyo, Japan; ^4^School of Veterinary Medicine, Nippon Veterinary and Life Science University, Musashino, Tokyo, Japan

**Keywords:** adiponectin, cat, lipid metabolism, obesity, resveratrol, serum amyloid A

## Abstract

**Introduction:**

The prevalence of lipid metabolism disorders, including obesity, increases with age in cats and humans. Obesity is a condition characterized by systemic low-grade inflammation and oxidative stress caused by excessive visceral fat accumulation. Resveratrol (RSV), a natural plant polyphenol, modulates the expression of anti-inflammatory factors. This study aimed to investigate the effects of resveratrol supplementation on lipid metabolism in both healthy and obese cats and assess its potential as a dietary supplement for improving lipid metabolism disorders in this population.

**Methods:**

Plasma metabolite and hormone concentrations, and enzyme activities were measured in healthy, obese, and overweight cats supplemented with RSV for 4 weeks. RVS was supplemented at 1 mg/kg body weight/day (low dose) and 5 mg/kg/day (high dose) in capsules for 4 weeks.

**Results:**

Body weight, body condition score, BUN, and insulin concentrations did not change in obese or overweight cats with RSV supplementation for 4 weeks. Plasma triglyceride, free fatty acids, and serum amyloid A (SAA) concentrations and lactate dehydrogenase (LDH) activities decreased, and adiponectin concentrations increased markedly in obese and overweight cats after RSV supplementation.

**Discussion:**

Decreased plasma SAA concentrations and LDH activities and increased plasma adiponectin concentrations in obese and overweight cats seem to be induced by the improvement in liver function and the anti-inflammatory effect of RSV. Moreover, RSV supplementation may be useful in treating lipid metabolism disorders, including obesity, in cats.

## 1 Introduction

The prevalence of lipid metabolism disorders, including obesity, increases with age in cats and humans (1, 2). Obesity is due to excessive triglyceride (TG) accumulation in the adipose tissue (AT) caused by an energy imbalance in which energy intake exceeds energy expenditure. When AT reaches its maximum capacity for energy storage, it releases free fatty acids (FFA), causing ectopic lipid deposition in other tissues, such as the liver, skeletal muscle, and vasculature. AT is characterized by increased macrophage infiltration during obesity development (3, 4). Consequently, AT macrophages secrete high levels of proinflammatory cytokines, resulting in obesity-associated chronic low-grade inflammation and impaired insulin signaling (5). Obesity and insulin resistance change with advancing age and are linked to chronic low-grade inflammation, leading to age-related systemic metabolic dysfunction, physical limitation, and frailty (6).

Cats are more prone to obesity than dogs because of their unique glucose and lipid metabolic characteristics (7, 8). In feline livers, glucokinase, the rate-limiting enzyme in glycolysis, is lacking (7), and gluconeogenic enzyme activities are higher than that in canine livers (9). Additionally, the expression levels of mRNA associated with insulin signaling pathway, including insulin receptor substrate (IRS)-1, IRS-2, phosphatidylinositol 3-kinase (PI3K) P-85 α, are significantly lower in cats than those in dogs (8). Furthermore, the secretion of adiponectin, an adipokine that improves insulin sensitivity, is lower in cats in the normal state (8) and with weight gain (10).

Obesity is a condition characterized by systemic low-grade inflammation and oxidative stress caused by excessive visceral fat accumulation. Weight reduction through energy restriction and increased physical activity are key strategies for obesity prevention (11). Moreover, the consumption of foods rich in bioactive anti-inflammatory compounds such as polyphenols has been shown to reduce inflammation (12). Resveratrol (RSV), a natural polyphenol found in plants such as peanuts, grapes, and strawberries (13), modulates the expression of pro-and anti-apoptotic factors, neutralizes free radical species, affects mitochondrial function, chelates redox-active transition metal ions, and prevents protein aggregation (14).

This study aimed to investigate the effects of resveratrol supplementation on lipid metabolism in both healthy and obese cats and assess its potential as a dietary supplement for improving lipid metabolism disorders in this population.

## 2 Methods

### 2.1 Animals

Cats are kept in two branches, Nabiya Sarang Ha and Dong Go Dong Rak, of Korean Private Shelters (Seoul, South Korea). Nabiya Sarangha Branch has 300 cats (70% male, 30% female; domestic short-haired cats, 2–11 years old), and Dong Go Dong Rak Branch has 150 cats (80% male, 20% female; domestic short-haired cats, 2–113 years old). All the cats examined were kept individually in cages and provided the same condition for 4 weeks with environmental maintained at 25.0 ± 2.0°C and 55.0 ± 10.0% relative humidity, and on a 13:11h, light: dark cycle (light on 8 a.m. to 9:00 p.m.). All cats were fed with a commercial diet, Royal Canin Feline Health Nutrition Indoor 27 Cat Dry Food (Royal Canin Korea, Seoul, South Korea), two times a day at 9:00 a.m. and 8:00 p.m. and free access to water. Body weight (BW) and body condition score (BCS) were measured before (week 0) and 4 weeks after supplementation (week 4). BCS was assessed using a 5-point scale system (15, 16) as follows: BCS1, very thin; BCS2, underweight; BCS3, ideal; BCS4, overweight; BCS5, obese. A total of 12 clinically healthy cats (domestic short-haired cats, six females and six males, aged 3–7 years) and 4 obese cats (domestic short-haired cats, three males and one female, aged 7–8 years) were used in this study.

### 2.2 Resveratrol supplementation

Healthy cats were divided into three groups: control without RVS supplementation (2 males, 2 females; 4–6 years old), RSV low-dose group (1 mg/kg body weight/day; 2 males, 2 females; 4–6 years old), and RSV high-dose group (5 mg/kg/day; 2 males, 2 females; 3–5 years old). Powdered RSV (Trans Resveratrol Powder Pure Bulk) was purchased from PureBulk Inc. (Roseburg, OR, USA), and packed in capsules (Suheung Capsule Co., Ltd., Seoul, South Korea). Capsule filled with and without the required amount of powdered RSV were administered orally to each cat with diet at 9:00 a.m. every day for 4 weeks.

### 2.3 Blood sampling and metabolite, hormone, and enzyme assay

A total of 1.5 mL of blood was collected from the jugular vein of each animal into heparinized tubes before (0 weeks) and 4 weeks after RVS supplementation. Blood collection was performed before the morning feeding and the collected samples were centrifuged at 2,000 × g for 5 min at 4°C to obtain plasma. The plasma samples were stored at−25°C until use. Plasma glucose, TG, total cholesterol (TC), blood urea nitrogen (BUN), creatinine, alanine aminotransferase (ALT), and lactate dehydrogenase (LDH) activities were measured using an autoanalyzer (Catalyst One, IDXX, Westbrook, Maine 04092 USA). Plasma-free fatty acid (FFA) and serum amyloid A (SAA) concentrations were measured using the NEFA Test Wako (Wako Pure Chemical, Tokyo, Japan) and VET-SAA kits (Eiken Chemical Co., Tokyo, Japan), respectively. The SAA concentration is an inflammatory marker in cats (17). Adiponectin and insulin concentrations were measured using commercial ELISA kits a mouse/rat adiponectin ELISA kit (Otsuka Pharmaceutical Co., Ltd., Tokyo, Japan) and a rat insulin ELISA kit (AKRIN-010T; Shibayagi Co., Gunma, Japan), respectively.

### 2.4 Statistical analysis

All measured values are expressed as means ± standard deviation (SD). Statistical significance was determined using the paired *t*-test. The significance level was set at *p* < 0.05. Statistical analyses were performed using Microsoft Excel.

## 3 Results

Table 1 shows the changes in metabolite and hormone concentrations and enzyme activities in the plasma of healthy cats supplemented with RSV for 4 weeks. All values measured in healthy control cats without RSV supplementation were maintained within the reference values for 4 weeks. BW, BCS, glucose, TG, TC, BUN, creatinine, insulin concentrations, and ALT activities in healthy cats (low-dose and high-dose groups) did not change after 4 weeks of RVS supplementation. Plasma FFA and SAA concentrations and LDH activities decreased, whereas plasma adiponectin concentrations increased significantly in the RSV low-dose group. These changes were not in dose-dependent manner upon RSV supplementation in healthy cats.

**Table 1:** Changes in fasting plasma metabolites and hormone concentrations and enzyme activities in healthy cats supplemented with resveratrol.

**Group**	**Control without RSV**		**Low dose of RSV**		**High dose of RSV**	
	**0 week**	**4 week**	**0 week**	**4 week**	**0 week**	**4 week**
Body weight (kg)	4.9 ± 0.9^§^	4.8 ±0.8	5.2 ± 0.4	5.0 ± 0.4^§^	5.1 ± 0.7^§^	5.1 ± 0.6
Body condition score	3.3 ± 0.4	3.3 ± 0.4	3.5 ± 0.5	3.5 ± 0.5	3.3 ± 0.4	3.3 ± 0.4
Glucose (mg/100 mL)	72 ± 18^§^	80 ± 10^§^	66 ± 8^§^	82 ± 13^§^	82 ± 16^§^	96 ± 19
Triglyceride (mg/100 mL)	68 ± 23	61 ± 21	59 ± 5	54 ± 4	74 ± 10	73 ± 12
T-cholesterol (mg/100 mL)	142 ± 27	141 ± 26	139 ± 6	145 ± 7	144 ± 15	152 ± 11
FFA (mEq/L)	0.7 ± 0.1	0.7 ± 0.1	0.8 ± 0.1	0.6 ± 0.1	0.8 ± 0.1	0.7 ± 0.2
BUN (mg/100 mL)	20 ± 2^§^	17 ± 3	21 ± 5	19 ± 4	21 ± 2	20 ± 2
Creatinine (mg/100 mL)	1.4 ± 0.1	1.3 ± 0.3	1.3 ± 0.1	1.2 ± 0.2	1.4 ± 0.2	1.3 ± 0.1
ALT (IU/L)	35 ± 8	37 ± 10	43 ± 11	39 ± 10	44 ± 10	42 ± 8
LDH (IU/L)	502 ± 147	367 ± 36	651 ± 311	431 ± 167	735 ± 134	479 ± 175
SAA (mg/L)	1.3 ± 0.4	1.1 ± 0.3	0.9 ± 0.1	1.0 ± 0.8	1.4 ± 0.9	0.6 ± 0.3
Adiponectin (μg/mL)	1.7 ± 0.3	1.6 ± 0.4	1.7 ± 0.2	2.2 ± 0.2^*^	1.4 ± 0.1	1.9 ± 0.6
Insulin (ng/mL)	0.6 ± 0.1	0.6 ± 0.1	0.7 ± 0.1	0.9 ± 0.2	0.7 ± 0.1	0.8 ± 0.1

^§^Values are means ± standard deviation (SD).

^*^Significantly different from the 0 week values in the same group (p < 0.05).

FFA, free fatty acid; SAA, serum amyloid A; ALT, alanine aminotransferase; LDH, lactate dehydrogenase.

Table 2 shows the changes in metabolite and hormone concentrations and enzyme activities in the plasma of obese and overweight cats supplemented with RSV. BW, BCS, BUN, and insulin concentrations did not change in obese and overweight cats supplemented with RSV at 5 mg/kg/day (high dose) for 4 weeks. Plasma glucose concentrations were increased in three cats and creatinine concentrations were decreased in all four cats. Plasma TG, FFA, and SAA concentrations and LDH activities decreased remarkably in the four cats after RSV supplementation. In contrast, the plasma adiponectin concentrations increased remarkably in the four cats after RSV supplementation.

**Table 2:** Changes in fasting plasma metabolites and hormones concentrations and enzyme activities in obese cats supplemented with resveratrol.

	**Obese 1**		**Obese 2**		**Obese 3**		**Overweight 1**	
	**0 week**	**4 week**	**0 week**	**4 week**	**0 week**	**4 week**	**0 week**	**4 week**
Age (years old)/Sex	8/male		7/male		7/male		7/female	
Body weight (kg)	6.9^§^	7.3	8.1	8.2	6.1	6.0	5.6	5.8
Body condition score	5	5	4.5	4.5	4.5	4.5	4	4
Glucose (mg/100 mL)	90^§^	53^§^	136	180	67	72	128	154
Triglyceride (mg/100 mL)	376	312	359	305	142	99	375	211
T-cholesterol (mg/100 mL)	182	179	157	149	119	140	124	150
FFA (mEq/L)	1.4	0.7	1.2	0.8	1.0	0.4	1.4	0.8
BUN (mg/100 mL)	20	23	18	21	14	13	22	22
Creatinine (mg/100 mL)	1.8	1.2	1.8	1.6	1.5	1.2	1.7	1.1
ALT (IU/L)	58	39	32	31	34	39	58	70
LDH (IU/L)	872	326	3,090	236	1,026	293	1,306	410
SAA (mg/L)	0.9	0.4	1.8	0.6	2.6	0.9	2.3	1.3
Adiponectin (μg/mL)	0.8	0.9	0.8	1.2	0.8	1.5	0.8	2.1
Insulin (ng/mL)	0.6	0.7	0.8	0.7	0.8	0.7	0.9	0.7

^§^Obese and overweight cats were supplemented with RSV at 5 mg/kg/day for 4 weeks.

## 4 Discussion

This study aimed to investigate the effects of resveratrol supplementation on lipid metabolism in both healthy and obese cats and assess its potential as a dietary supplement for improving lipid metabolism disorders in this population. Remarkable changes in LDH activities and an increase in adiponectin concentrations were observed in healthy cats supplemented with RSV. TG and FFA concentrations were maintained within the reference ranges. These results indicate that RSV supplementation improves liver function and lipid metabolism in cats. The response to RSV supplementation was not in dose-dependent manner. In obese and overweight cats, RSV supplementation markedly improved lipid metabolism (decreased plasma TG and FFA concentrations) and exerted anti-inflammatory effects (decreased plasma SAA concentration). These changes are thought to be due to liver function improvement (decrease in plasma LDH activity) and an increase in plasma adiponectin concentrations.

Resveratrol has anti-inflammatory (18), antioxidant (19), anti-obesogenic (20, 21), and anti-diabetic effects (22, 23). Furthermore, RSV is hypothesized to be potentially useful in protecting against factors that increase the susceptibility to metabolic syndrome (24, 25). A moderate dose of resveratrol activates Sirtuin 1 (NAD-dependent deacetylase sirtuin-1) followed by activating AMP activated protein kinase (AMPK), whereas a high dose of RSV activates AMPK in a Sirtuin 1-independent manner (26). AMPK activation induces amelioration of glucose and lipid metabolism in livers resulting in reduction of glucose and TG concentrations in obese animals (27, 28). RSV supplementation induces decreasing in the plasma glucose and TG concentrations in obese animals, but in cats supplemented with RSV, plasma glucose concentrations increased in this study. This change was not statistically significant. On the other hand, in some types of cancer cells, Sirtuin 1 activation is able to inhibit glucose uptake (29). The reason of increased plasma glucose concentrations observed in cats supplemented with RSV should be further studied in many cats including expression of associated genes.

In obese and overweight cats, a clear improvement in liver function, such as a decrease in LDH activity, was observed after RSV supplementation. RSV supplementation ameliorates liver function in animals with severe liver dysfunction like non-alcoholic fatty liver disease (NAFLD) (30, 31). Changes in cholesterol concentrations and ALT activities may associate with liver function improvement caused by RSV supplementation. Moreover, there are many reports that RSV supplementation improves hypertension and kidney dysfunction in rodents with chronic kidney disease (CKD) (32, 33). RSV supplementation may ameliorate kidney function leading decreased plasma creatinine concentrations in the obese cat.

RSV supplementation increases plasma adiponectin concentrations in obese and overweight cats. Obesity induces adipocyte senescence followed by a senescence-associated secretory phenotype (SASP) like decreased adiponectin secretion, which triggers a cascade of reactions leading to inflammation, insulin signaling disruption, insulin resistance, and severe metabolic disorders (34, 35). In contrast, RSV shows potent anti-SASP and anti-inflammatory activities (36, 37) and improves hepatic function (38). Decreased plasma SAA concentrations and LDH activities and increased plasma adiponectin concentrations in obese cats seemed to be induced by the improvement in liver function and the anti-SASP effect of RSV (39). Moreover, RSV supplementation may be useful for treating lipid metabolism disorders, including obesity, and may be effective in preventing age-related diseases in cats.

This study is preliminary, and its limitations include the small number of samples and biological and environmental variables (age, sex, diet, and others). The optimal dose and duration of RSV supplementation for cats have not been sufficiently examined. More animal studies are needed to assess the precise effect of RSV supplementation. In particular, long-term effect of RSV supplementation in obese cats should be investigated in detail in animals with different severities of obesity. Moreover, further studies are needed to evaluate the usefulness of RSV as anti-obesity supplement in a large number of animals.

## 5 Conclusion

Healthy, obese, and overweight cats were supplemented with RSV for 4 weeks. Plasma metabolite and hormone concentrations, and enzyme activities were measured in cats before (0 weeks) and 4 weeks after RSV supplementation. RVS was administered as a capsule at a dose of 1 mg/kg body weight/day (low-dose group) or 5 mg/kg/day (high-dose group) for 4 weeks. Body weight, BCS, BUN, and insulin concentrations did not change in obese or overweight cats with RSV supplementation (high dose, 5 mg/kg/day) for 4 weeks. Plasma TG, FFA, and SAA concentrations, and LDH activity decreased remarkably in obese and overweight cats after RSV supplementation. Plasma adiponectin concentrations increased markedly in all four cats after RSV supplementation. Decreased plasma SAA concentrations and LDH activity and increased plasma adiponectin concentrations in obese and overweight cats seemed to be induced by the improvement in liver function and the anti-SASP effect of RSV. Moreover, RSV supplementation may be useful in the treatment of lipid metabolism disorders, including obesity, in cats.

## Data Availability

The raw data supporting the conclusions of this article will be made available by the authors, without undue reservation.
